# Impact of the Morphology of Electrospun Lignin/Ethylcellulose Nanostructures on Their Capacity to Thicken Castor Oil

**DOI:** 10.3390/polym14214741

**Published:** 2022-11-05

**Authors:** María Borrego, José E. Martín-Alfonso, Concepción Valencia, M. Carmen Sánchez, José M. Franco

**Affiliations:** Chemical Product and Process Technology Research Center (Pro2TecS), Department of Chemical Engineering and Materials Science, ETSI, Campus de “El Carmen”, University of Huelva, 21071 Huelva, Spain

**Keywords:** lignin, ethylcellulose, electrospinning, nanostructure, dispersion, rheology

## Abstract

This study reports on a novel strategy for manufacturing thickened gel-like castor oil formulations by dispersing electrospun lignin/ethylcellulose nanostructures. These thickened formulations were rheologically and tribologically evaluated with the aim of being proposed as alternative ecofriendly lubricating greases. Low-sulfonate kraft lignin (LSL) and ethylcellulose (EC) were dissolved in a DMAc:THF mixture at different concentrations (8, 10, and 15 wt.%) and LSL:EC ratios (50:50, 70:30, and 90:10) and subjected to electrospinning. The resulting electrospun nanostructures were morphologically characterized. EC acting as the cospinning polymer improved both LSL spinnability and the oil structuring ability. Solutions with a high lignin content achieved microsized particles connected by fibrils, whereas solutions with a high EC content (50:50 ratio) and LSL/EC total concentration (10 and 15 wt.%) yielded beaded or bead-free nanofibers, due to enhanced extensional viscoelastic properties and nonNewtonian characteristics. The gel-like properties of electrospun nanostructure dispersions in castor oil were strengthened with the nanostructure concentration and the EC:LSL ratio, as a result of the formation of a more interconnected fiber network. The oleodispersions studied exhibited a satisfactory frictional response in a tribological contact, with friction coefficient values that were comparable to those achieved with traditional lithium-lubricating greases.

## 1. Introduction

Biopolymer-based micro- and nanofibers are increasingly being introduced into different types of materials and formulations for a wide range of applications owing to their outstanding properties, such as high specific surface area, high porosity, and superior stiffness and tensile strength as compared to conventional fibers, and ease of functionalization [1]. Filtration in environmental and energy fields [2], food packaging [3], catalysis [4], biomedical [5], and many others [6,7] are included among the wide range of applications. Different methods are available to generate polymeric micro- and nanofibers at present, for instance, phase separation [8], drawing [9,10], template synthesis [11], self-assembly [12], and electrospinning [13]. Among all of them, electrospinning may be considered the simplest one for nanofiber production. Electrospinning has emerged as a simple and cost-effective method to produce nanofibers from a variety of polymer sources. As is well known, electrospinning refers to the formation of fibers from a polymer solution, which is ejected from a thin spinneret located between two electrodes with opposite polarity electrical charges [14], one placed onto the spinneret and the other onto a fiber collector. The charged solution jet evaporates on its way to the collector typically to form an arrangement of nonwoven fibers. The potential difference may be modified to achieve the required morphology, depending on the physicochemical properties of the solution, i.e., electrical conductivity, surface tension, and viscosity [15]. Electrospinning processing parameters, such as electrodes spacing, voltage, humidity, volumetric feed flow, and needle diameter, may also be tuned to control solvent evaporation, preventing fibers from melting or failing to form [6,16]. A wide variety of biopolymers, such as proteins, polysaccharides, and lignocellulosic biomass components, have been used to produce renewable nanofibers [17,18] with tailored functional properties that can be achieved by controlling the intermolecular interactions among polymeric molecules.

Lignocellulosic biomass is basically composed of cellulose, hemicellulose, and lignin as the main structural biopolymers. Lignin is especially interesting as it is composed of aromatic structures, which have the potential to replace aromatic polymers and fine chemicals of industrial interest [19,20]. The primary structure of lignin is formed by copolymerized three main phenylpropane monomers, i.e., sinapyl, coniferyl, and *p*-coumaryl alcohols [21]. Roughly, 50 million tons of residual lignin are annually produced by the paper industry, but only ~2% has been commercially employed in low-value chemicals, such as dispersants, adhesives, or surfactants [22,23]. Electrospinning of pure lignin is challenging due to its structural complexity and variability of the composition, according to the different processes and different biomass resources, which cause a potential lack of molecular entanglement, leading to a failure in fiber formation [24]. Aiming to overcome these problems, several polymers, such as polyvinyl acetate (PVA) [25,26], poly ethylene oxide (PEO) [27,28], polyhydroxybutyrate (PHB) [29], polycaprolactone (PCL) [30,31], polylactic acid (PLA) [32], and polyvinylpyrrolidone (PVP) [33] have been incorporated to lignin solutions in order to improve their electrospinnability. Most of the studies conducted to date were focused on blends of lignin with synthetic polymers, which might be expected to behave differently from blends with other natural polymers or derived from these, such as ethylcellulose. The production of ethylcellulose electrospun micro- or nanostructures, rather than with synthetic polymers, may also be interesting from the point of view of compatibility with nonpolar fluids because of their hydrophobic–oleophilic character [34], which could give rise to porous structures where oils can be adsorbed or entrapped, and therefore to stimulate and promote oil structuring.

Oil structuring is generally referred to as oleogelation and is applied to obtain soft and semisolid oil-based products [35]. The use of thickening agents to form gels from vegetable oils has been extensively studied [36,37] in the last years. With continuous growth in this field, different thickeners and vegetable oils have been explored to produce oleogels. The performances, applications, and properties of oleogels depend on the nature of their components (vegetable oil/gelator) and the microstructure (kind of assembly, crystal structure, morphology, crystallinity, etc.) achieved during the manufacturing process [38]. In the most common oleogelation process, the gelator molecules led to the formation of oleogel through the direct dispersion method. This process generally involves mixing gelators in the vegetable oil at a temperature higher than their melting point. This step is followed by a cooling step where a three-dimensional network formation occurs that entraps the oil. Other indirect methods to obtain gel oils have been proposed, such as the so-called foam-templated [39] and emulsion-templated [40] approaches or stepwise solvent-exchange [41] routes, yielding porous nanostructures where oil can be adsorbed or entrapped. However, most of these methods are tedious and very time- and/or energy-consuming. Thus, alternative strategies for oleogelation and oil structuring might attract great interest in several engineering domains and products, such as pharmaceuticals, food industry, and lubricants. In particular, in the lubricant field, obtaining oleogels from natural and renewable components is challenging in terms of the substitution of products derived from petrochemicals.

Taking into account these considerations, this study reports a novel strategy for manufacturing gel-like oleo-dispersions based on electrospun lignin/ethylcellulose nanostructures and castor oil. First, the electrospinnability of low-sulfonate lignin solutions in a DMAc:THF solvent was evaluated using ethylcellulose as a cospinning polymer. The morphological properties of the nanostructures were related to the physicochemical properties of the solutions. Then, the nanostructures were used as thickening agents to promote the structuring of castor oil, assessing the rheological and tribological properties of these gel-like dispersions aiming to explore their potential as lubricants.

## 2. Materials and Methods

### 2.1. Materials

Softwood low-sulfonate kraft lignin (LSL, Mw: ~10 kDa) and ethylcellulose (EC, Mw: ~82 kDa, 48% ethoxyl) from Merck Sigma-Aldrich (Taufkirchen, Germany) were used as base materials for the preparation of the electrospun structures. *N,N*-dimethylacetamide (DMAc, purity ≥99.8%) and tetrahydrofuran (THF, purity ≥99.0%) from Sigma-Aldrich were used as solvents to prepare LSL/EC solutions. Castor oil (211 cSt at 40 °C) was supplied by Guinama (Valencia, Spain) and used to prepare the oleo-dispersions.

### 2.2. Preparation and Characterization of Polymer Solutions for Electrospinning

LSL/EC biopolymers were dissolved in a DMAc:THF (1:1 wt/wt) blend to attain 8, 10, and 15 wt.% total concentrations, modifying the LSL:EC ratio (50:50, 70:30, and 90:10). This binary solvent was selected on the basis of the proper ability to solve EC for electrospinning purposes studied in a previous work [42]. First, EC solutions were prepared by magnetic stirring (300 rpm) in the DMAc:THF binary solvent during 2 h, at 40 °C. Then, LSL was added to the EC solution and left under agitation for 24 h. Finally, solutions were transferred to centrifuge tubes and centrifuged at 3000 rpm for 10 min to remove any remaining impurities from the solution.

Physicochemical characterization of the polymeric solutions was carried out by means of electrical conductivity, surface tension, and shear and extensional viscosity measurements.

Electrical conductivity of LSL:EC solutions was tested in a Crison (GLP 31) conductivity meter (Crison, Barcelona, Spain). The measurements were performed at 23 °C and reported after five times replication.

Surface tension of the different solutions was measured using a dynamic Wilhelmy plate tensiometer Sigma 703D (Biolin Scientific, Beijing, China) at 20 °C and repeated five times.

Measurements of shear viscosity were performed at 23 °C in an ARES-controlled strain rheometer (Rheometric Scientific, Leatherhead, UK) using a conventional coaxial cylinder geometry (32 mm inner diameter, 2 mm gap, 33.35 mm length) in a 0.03-300 s^−1^ shear rate range. Some LSL/EC solutions exhibited a non-Newtonian response, where the shear rate dependence of viscosity was described well by the Williamson model (R^2^ < 0.995):

(1) $$\eta =\frac{\eta_{0}}{1+{\left(k\cdot \dot{\gamma }\right)}^{m}}$$

where *η* is the non-Newtonian viscosity; $\dot{\gamma }$ is the shear rate; *η*_0_ is the zero-shear-rate-limiting viscosity; *m* is the parameter related to the slope of the shear-thinning region, and *k* is the constant whose inverse coincides with the shear rate for which *η* = *η*_0_/2.

Extensional viscosity of solutions was measured using the CaBER-1 capillary break-up extensional rheometer (ThermoHaake, Karlsruhe, Germany). The break-up of the fluid filament after stretching and the filament diameter vs. time evolution were measured with a laser micrometer. To perform tests, 6 mm parallel plates were set at an initial plate separation of 1 mm (h_0_). Then, a given strain was suddenly imposed to create a filament separating the plates from their initial distance h_0_ to their final separation h_f_  = 10.0 mm within 40 ms at a constant separating speed. Once the fluid filament was formed between the plates, it drained through capillary action depending on the elastic relaxation time of the fluid. The extensional rheological properties were indirectly deduced from the evolution of the filament diameter with time, and the extensional viscosity was quantified as:

(2) $$\eta_{ext}=\frac{\sigma }{\left(-\frac{dD\left(t\right)}{dt}\right)}$$

where *η_ext_* is the extensional viscosity; *σ* is the surface tension of the polymer solution; *D* is the diameter of the filament, and t is the time. Three replicates of each extensional test were performed on fresh samples.

### 2.3. Electrospinning Process

LSL/EC solutions were electrospun using an in-house electrospinning apparatus, consisting of a high-voltage power supply (Spellman, Hauppauge, NY, USA), a syringe with a blunt metal needle, a syringe pump (KD Scientific Inc.; Holliston, MA, USA, and an electrically grounded collector. The LSL/EC solutions were pumped at a specified speed (0.8 mL/h) through the needle tip while applying a high voltage (20 kV). Tip-to-target distance was 12 cm, and tip diameter was ≈ 0.6 mm. All the electrospinning experiments were carried out at room temperature (~ 23 °C) and relative humidity (~ 55%).

### 2.4. Morphological Characterization of Nanostructures

The morphology of LSL/EC nanostructures was observed in a FlexSEM 1000 II microscope (Hitachi, Tokyo, Japan) at an accelerating voltage of 20 kV. Each sample was coated with gold for analysis. The average diameters of the obtained electrospun fiber mats were determined by using image-analytical software (Image J; NIMH, Bethesda, MD, USA).

### 2.5. Preparation and Characterization of Dispersions of Electrospun Nanostructures in Castor Oil

LSL:EC nanostructures produced by electrospinning were dispersed in castor oil using an open vessel with an anchor impeller geometry at room temperature (~23 °C) and a rotation speed of 60 rpm during 60 min, at different concentrations of (10, 20, and 30 wt.%). Then, the dispersions were stored at least 24 h to carry out the rheological and tribological characterizations. The homogeneity of the resulting dispersions was verified through optical observations and digital photos.

Rheological characterization of oleo-dispersions was conducted in a Rheoscope controlled-stress rheometer (ThermoHakee, Karlsruhe, Germany), using roughened stainless steel parallel plate geometries (20 and 35 mm, 1 mm gap). Small-amplitude oscillatory shear (SAOS) tests were performed inside the linear viscoelastic region, in a frequency range comprised between 0.03 and 100 rad/s. Stress sweep tests were previously performed to determine the linear viscoelastic regime. At least two replicates were conducted for every sample. The tribological performance of the oleo-dispersions was investigated in a tribological cell coupled to a MCR-501 rheometer (Anton Paar, Graz, Austria) consisting of a 6.35 mm diameter steel ball rotating on three 45°-inclined rectangular steel plates, on which the samples to be tested as lubricants were spread. Constant normal load and rotational speed of 30 N and 30 min^−1^, respectively, were applied for 10 min. Testing time was long enough to achieve stationary values of the friction coefficient. At least five replicates were made for each oleo-dispersion sample. The wear scars produced on the steel plates were analyzed using a BX51 microscope (Olympus, Tokyo, Japan), from which the mean diameters were determined.

## 3. Results and Discussion

### 3.1. Physicochemical Properties of LSL:EC Solutions

The electrical conductivity, surface tension, and shear and extensional rheological properties of the LSL:EC solutions in DMAc:THF were measured, and the results are shown in Table 1. As can be observed, EC (LSL-free) solutions displayed very low values of electrical conductivity (33.53–30.81 µS/cm), and this property increased with the total polymer concentration, but particularity with the LSL content. This fact could be related to the polar character of lignin, due to the presence of phenolic and aliphatic hydroxyl and carboxyl groups as well as substructures based on β-O-4′alkyl ethers in its chemical structure [19]. In addition, the increase in conductivity with the LSL concentration suggests that these solutions are below the concentration at which the macromolecules start to overlap (overlap concentration), delimiting the transition between the dilute and semidilute regime, from which the conductivity decreases due to the reduced mobility of the entangled polymeric molecules [43]. Moreover, the higher the lignin proportion in the blend, the lower the surface tension of the LSL:EC solutions (see Table 1). A similar behavior was found by Ago et al. [26] for lignin–PVA blends and by Borrego et al. [44] for lignin/PVP–surfactant mixtures. This fact could represent a positive point with respect to the fiber-forming ability of LSL:EC solutions. However, the balance with the other important properties, such as viscosity and electrical conductivity, should be taken into account in the right fiber formation.

**Figure 1:**
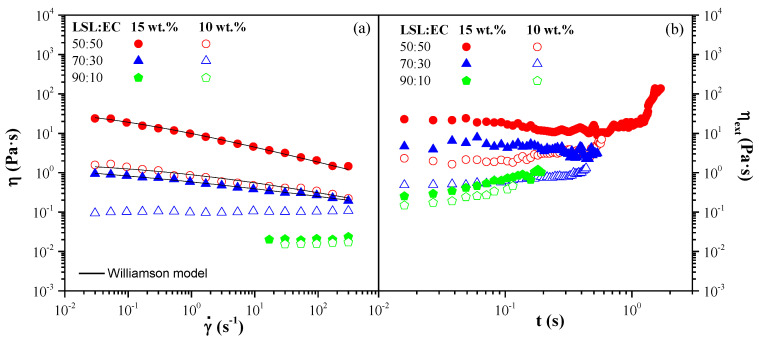
(**a**) Viscous flow curve of LSL:EC solutions in DMAc:THF to 15 and 10 wt.% total concentration. Solid lines represent fits to the Williamson model with the parameters given in Table 1 and (**b**) Extensional viscosity as a function of time.

Figure 1a shows the viscosity curves for solutions with 10 and 15 wt.% total concentrations and variable LSL:EC ratios. According to the experimental results of shear rheology, most of the solutions containing 8 and 10 wt.% total concentrations of the LSL:EC blends showed Newtonian behavior throughout the whole shear rate range studied, with apparent viscosity values that decreased with the LSL content. It is well known that the three-dimensional structural network of lignin particles is composed of aromatic monolignols, and these molecular units are not able to easily produce physical entanglements like linear polymers, such as EC. Thus, an increase in LSL content resulted in lower viscosity values of the LSL:EC solutions. However, at a higher LSL:EC concentration (15 wt.%, except for the 90:10 LSL:EC ratio) the shear flow response became shear thinning, with a tendency to reach a Newtonian plateau at low shear rates (at around 0.1 s^−1^). The shear rate dependence of viscosity fitted the Williamson model (see Figure 1a) well, and the parameters obtained from the fitting to this model are shown in Table 1. As can be observed, the η_0_ and shear-thinning character (*m*) decrease as the LSL content increases, while *k* values increase.

Regarding the extensional rheology, the extensional viscosity of solutions with 8 wt.% total LSL:EC concentration and that containing 10 wt.% polymers at a 10:90 LSL:EC ratio could not be experimentally measured since the filament ruptured immediately after imposing the stretching strain as a result of their poor elastic properties. Nevertheless, the extensional properties of the LSL:EC solutions with higher concentrations were satisfactorily assessed. Since capillary extensional processes are inherently transient phenomena, the extensional viscosity continuously increases when moving from the coiled to stretched states [45]. A convenient parameter accounting for the whole filament thinning process is the characteristic relaxation time (*λ*). For a polymer solution, the decay of the filament diameter *D*(*t*) respecting the initial value *D*_0_, i.e., just after imposing the strain, can be described as [46]:

(3) $$\frac{D\left(t\right)}{D_{0}}=A\;exp\left(-\frac{t}{3\lambda }\right)\;\;$$

where *λ* is the relaxation time, and *A* is the fitting parameter, which is proportional to the elastic modulus. The characteristic relaxation time *λ* was extracted from the fitting of the experimental *D*(*t*) vs. time data to Equation (3), and the resulting values are shown for each LSL:EC solution in Table 1 as well as the values of the fitting parameter *A*. The transient extensional viscosity can be calculated directly from the diameter decay according to:

(4) $$\eta_{ext}=-\frac{\sigma }{dD\left(t\right)/dt}\;\;\;\;$$

The extensional viscosity is displayed as a function of time in Figure 1b. As can be seen, the extensional viscosity values are constant for a very short time scale and subsequently increase monotonically until filament breakup. The short time-limiting extensional viscosity values (*η*_*ext*,0_) are also provided in Table 1. As expected, both the characteristic relaxation time and the apparent extensional viscosity increase with the total LSL:EC concentration and decrease by increasing the LSL:EC ratio. 

### 3.2. Electrospinnability of LSL:EC Solutions

**Figure 2:**
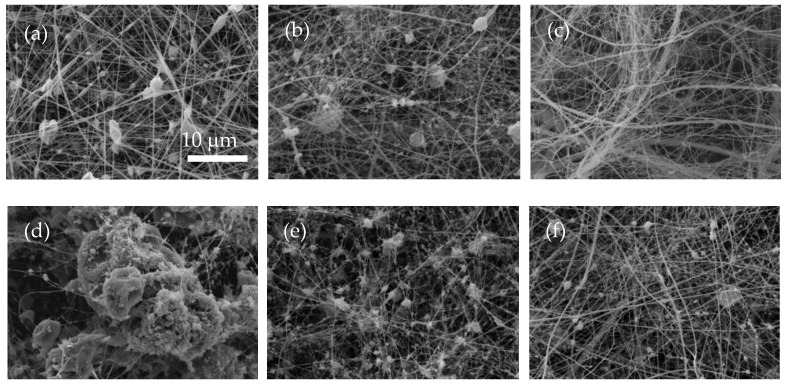

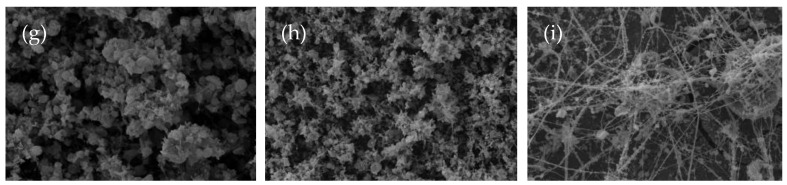
SEM images of electrospinning obtained from LSL:EC solutions at different concentrations and LSL:EC ratios: 50:50 LSL:EC ratio (**a**) 8% wt., (**b**) 10% wt, (**c**) 15% wt, 70:30 LSL:EC ratio, (**d**) 8% wt., (**e**) 10% wt., (**f**) 15% wt., 90:10 LSL:EC ratio, (**g**) 8% wt., (**h**) 10% wt, and (**i**) 15% wt.

Figure 2 displays the SEM images of the morphology of the resulting LSL:EC nanostructures obtained by electrospinning for different LSL:EC weight ratios (50:50, 70:30, and 90:10 wt/wt) and total polymer concentrations (8, 10, and 15 wt.%), respectively. As can be clearly inferred from SEM images (Figure 2), the composition of the spinning solutions had a significant impact on the morphology of the resulting nanostructures. For a given LSL:EC solution concentration, a transition from microsized particles connected by fibrils to well-developed electrospun fiber networks was obtained by decreasing the LSL:EC weight ratio in the solution. Therefore, increasing the solution concentration and relatively decreasing the amount of LSL yielded a progressive transformation in the morphology of electrospun structures from agglomerated particles to a bead-on-string architectures or even almost bead-free nanofibers (see, for instance, Figure 2c). Regarding the relationship between the physicochemical properties of the LSL:EC solutions and the nanostructure morphology, it may be deduced that polymer solutions from which nanofibers that largely dominate the morphology are produced should preferentially be conductive, shear-thinning fluids and display extensional properties with relaxation times of at least around 30 ms. As we know, physicochemical properties of lignin solutions play a key role in achieving uniform fiber mats via electrospinning. According to Aslanzadeh et al. [27], a certain polymer fraction above the critical overlap concentration is required to obtain bead-free uniform nanofibers. On the other hand, Dallmayer et al. [28] reported the influence of the extensional properties of kraft lignin/PEO solutions, which were improved by increasing either the lignin concentration or the PEO/lignin ratio. Our results are in agreement with these findings. Bead-free fibers were obtained from the solution with the highest LSL/EC concentration (15 wt.%) and the lowest LSL:EC ratio (50:50), showing enhanced extensional properties. Moreover, nanofiber formation was favored when polymer solutions showed shear-thinning behavior, as a result of the easier filament stretching at the high spinning flow rate imposed. Table 2 shows the average fiber diameters of the obtained electrospun nanostructures. The average diameter of the nanofibers was in the range of 0.12–0.23 μm. In general, the diameter of the electrospun nanofibers slightly depended on both the polymer concentration and the LSL:EC weight ratio. Thinner nanofibers were obtained as the content of LSL increased or total polymer concentration decreased, which is also correlated with the number of beads or particles produced. As mentioned above, this fact can be associated with the poor rheological properties of the LSL-rich solutions (low viscosity and low or negligible elastic relaxation times), together with probable excessive conductivity, which favor higher elongation forces leading to a thinner fiber formation and/or fiber break-up.

### 3.3. Rheological and Tribological Properties of Dispersions of Electrospun Nanostructures in Castor Oil

**Figure 3:**
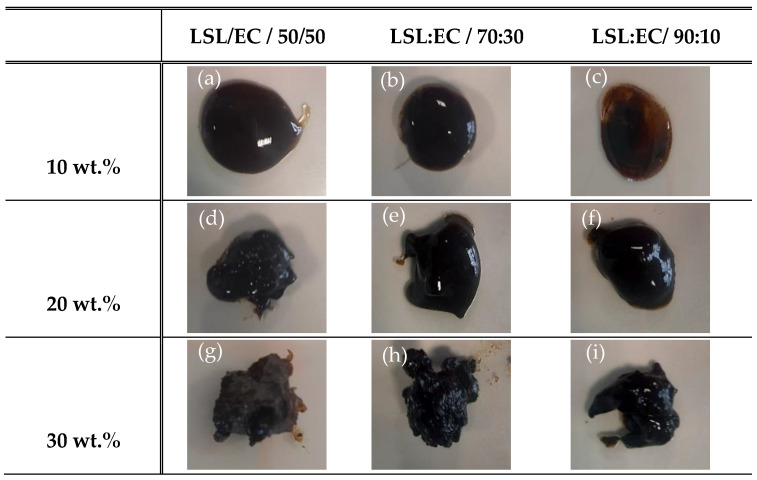
Physical appearance of LSL/EC nanostructures of different LSL:EC ratios (50:50, 70:30, 90:10) dispersed in castor oil at several concentrations (10, 20, and 30 wt.%).

In order to study the ability of the electrospun nanostructures to form gel-like oleo-dispersions, selected LSL:EC nanostructures obtained from the most concentrated solutions (15 wt.% total concentration of LSL/EC) were dispersed in castor oil by applying gentle mechanical agitation at different concentrations. For this study, these nanostructures were selected, since other electrospun samples, especially those obtained from the most diluted solutions (8 wt.%) and with a high LSL content, formed unstable dispersions in which phase separation was evidenced just after blending the components. Figure 3 shows the physical appearance of the LSL:EC nanostructures of different LSL:EC weight ratios (50:50, 70:30, and 90:10) dispersed in castor oil at several concentrations (10, 20, and 30 wt.%). As can be observed, all the nanostructures have a good oil-binding capacity and are physically stable against phase separation. However, the oleo-dispersion formulated with a higher LSL:EC ratio at 10 wt.% evidences signs of oil separation (see Figure 3c). These results confirm that homogeneous nanostructures largely dominated by nanofibers improved the interaction continuous phase–dispersed phase, giving rise to physically stable oleo-dispersions with a gel-like appearance. On the contrary, structures formed by particles or randomly distributed aggregates of particles interconnected by fibrils, led to unstable and/or slightly thickened liquid-like dispersions. Similar results were found for lignin/PVP nanostructures [33].

**Figure 4:**
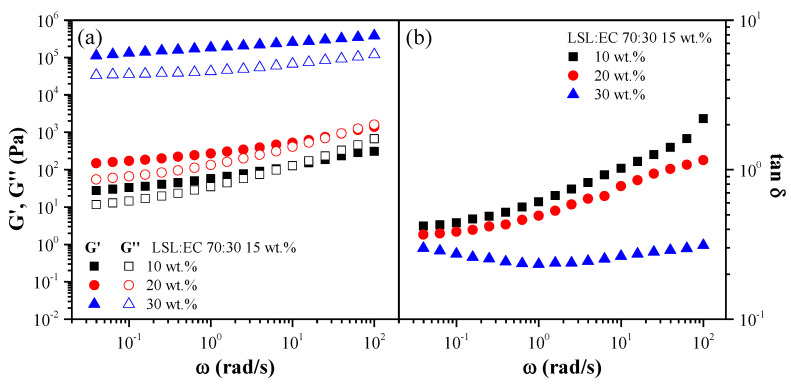
Frequency dependence of (**a**) the storage and loss moduli and (**b**) the loss tangent for oleo-dispersions formulated with the same nanostructure (70:30/LSL:EC ratio) at different concentrations.

**Figure 5:**
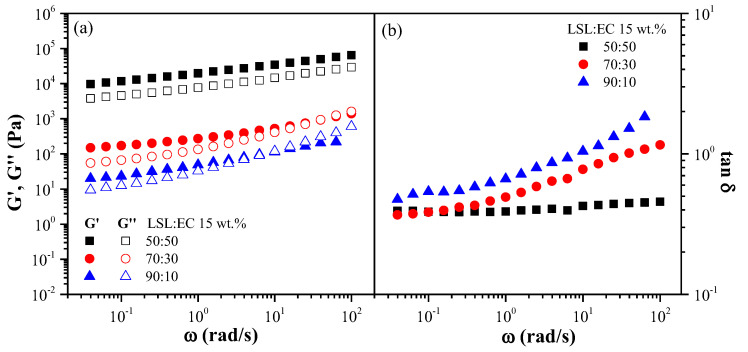
Frequency dependence of (**a**) the storage and loss moduli and (**b**) the loss tangent for oleo-dispersions formulated with nanostructures differing in the LSL:EC ratio (20 wt.% nanostructure concentration).

Figure 4 and Figure 5 display the evolution of SAOS functions, i.e., the storage (G’) and loss (G’’) moduli and loss tan (tan δ), with frequency, within the linear viscoelastic range, for selected oleo-dispersions as a function of the LSL:EC nanostructure concentration (Figure 4) and the LSL:EC ratio (Figure 5). As can be seen, oleo-dispersions formulated with a higher nanostructure concentration (30 wt.%) or 50:50 LSL:EC ratio showed a typical gel-like behavior where G’ > G’’ in the frequency range studied (tan δ < 1), where G’ and G’’ are only slightly dependent on ω [47]. However, a different behavior was found for dispersions with lower concentrations and higher LSL:EC ratios, since a crossover between G’ and G’’ at a high frequency occurred, and higher values of tan δ are observed. This crossover point corresponds to the end of the plateau region and to the beginning of the transition zone of the relaxation spectrum and decreased as the nanostructure concentration and EC content decreased. These results are indicative of a weaker gel-like response where a reduction in the extension of the plateau region takes place by lowering these variables. Similar mechanical spectra were obtained for oleo-dispersions formulated with electrospun ethylcellulose nanostructures [42].

**Figure 6:**
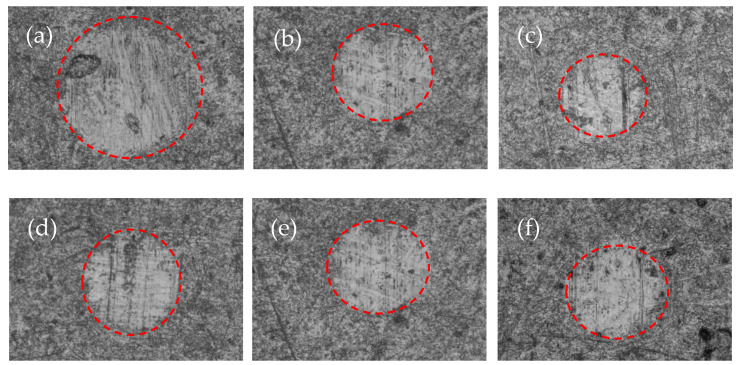
Wear scar micrographs of the lubricated plates with oleo-dispersions formulated with 70:30 LSL:EC nanostructures at different concentrations (**a**) 10 wt.%, (**b**) 20 wt.%, and (**c**) 30 wt.% and different LSL:EC ratios (**d**) 50:50, (**e**) 70:30, and (**f**) 90:10 and 20 wt.% nanostructure concentrations.

Aiming to explore the capacity of these oleo-dispersions based on the LSL/EC nanostructures and castor oil to act as semisolid lubricants, the tribological performance was evaluated in a ball-on-plates steel–steel tribological contact. The wear marks obtained on the plates after performing the tribological tests, i.e., after 10 min, are presented in Figure 6, and the corresponding average values of wear scar diameters and stationary friction coefficients are shown in Table 3. As can be observed, the contact surfaces of steel plates are damaged in all cases, and the rounded worn surfaces show a rather rough trace and deep furrows along the sliding direction (see Figure 6), suggesting a predominant abrasion wear mechanism. A decrease in the nanostructure concentration increased the wear scar size. Increasing the LSL:EC above 50:50 also tends to increase wear. These wear scar sizes were slightly lower than obtained with commercial mineral oil-based lithium grease [48]. On the contrary, very similar values of the friction coefficient, around 0.086 ± 0.008, were obtained in all cases, comparable to those found when using traditional lithium-lubricating greases or other gel-like dispersions based on synthetic polymers previously proposed as lubricants [49]. These results may be related to the rheological behavior, i.e., softer oleo-dispersions yield higher wear scar values, while oleo-dispersions formulated with higher nanostructure concentrations and a low LSL content exhibiting enhanced gel strength and significantly reduced wear. On the other hand, the friction coefficient values of these electrospun LSL:EC nanofiber-thickened oil formulations are comparable to that obtained for pure castor oil under similar conditions; however, the addition of nanofibers significantly improves the wear reduction [50].

## 4. Conclusions

In this work, electrospun nanostructures of low-sulfonate kraft lignin (LSL)/ethylcellulose (EC) were successful fabricated via electrospinning. EC was used as a cospinning polymer to improve the spinning stability and the fiber quality. The role of the spinning solution concentration and the LSL:EC ratio were systematically investigated. The morphology of the nanostructures obtained is specially influenced by the rheological properties of the solution, which depend on the total LSL/EC concentration and the LSL:EC ratio. Electrospun architectures composed of nanoparticles or microsized particles connected by fibrils were generated from solutions with lower LSL/EC concentrations and/or higher LSL:EC ratios. On the contrary, beaded fibers or uniform fiber networks were achieved by increasing the solution concentration and/or decreasing the LSL:EC ratio. This fact was mainly attributed to the shear and extensional rheological properties of LSL/EC solutions. In this sense, nanofiber-dominated morphologies were achieved from LSL/EC solutions with shear-thinning characteristics and relaxation times of at least 30 ms. Uniform fiber mats with some beads randomly distributed were obtained from the solutions with the lower LSL:EC ratios and higher LSL/EC concentrations, such as those having 15% wt. concentration and a 70:30 LSL:EC ratio or a 10% wt. concentration and a 50:50 LSL:EC ratio. Electrospun LSL/EC nanostructures formed by beaded nanofibers were able to form stable oleo-dispersions in castor oil, whereas structures characterized by nanoparticles or microsized particles connected by fibrils yielded unstable oleo-dispersions with a partial separation of phases. In general, the oleo-dispersions studied exhibited gel-like viscoelastic properties, with a crossover between G’’ and G’ in the medium–high frequency ranges studied, associated with the end of the plateau region, which was found for dispersions with higher LSL:EC ratios and lower nanostructure concentrations. On the contrary, a high nanostructure concentration and/or low LSL/EC ratio produced oleo-dispersions with higher gel stiffness that showed an extended plateau region in the mechanical spectrum. Thus, the viscoelastic properties of these oleo-dispersions may be tailored by modifying the nanostructure concentration and the LSL:EC ratio, according to the morphology of the nanostructure network achieved by electrospinning. The oleo-dispersions also demonstrated adequate tribological performance to be potentially applied as sustainable lubricating greases.

## Figures and Tables

**Table 1:** Electrical conductivity, surface tension, and shear and extensional viscosity of LSL:EC solutions in THF:DMAc.

Concentration (%.wt)	LSL:EC Ratio (%)	Electrical Conductivity, s (μS/cm)	Surface Tension, (mN/m)	Newtonian Viscosity, ƞ (Pa·s)	ƞ_o_ (Pa·s)	k (s)	m (-)	Extensional Viscosity ƞ_ext,o_ (Pa·s)	D_o_ (mm)	Relaxation time, λ (ms)	A (-)
8%	0:100 50:50 70:30 90:10	33.53 (±2.5 10^−1^) 75.01 (±2.3 10^−1^) 103.63 (±2.1 10^−1^) 108.20 (±1.0 10^−1^)	32.91 (±6.0 10^−1^) 28.21 (±1.0 10^−1^) 29.26 (±7.0 10^−1^) 28.89 (±1.5 10^−1^)	1.48 (±2 10^−1^) 1.2 × 10^−1^ (±3 10^−2^) 4.8 × 10^−2^ (±1 10^−2^) 4.5 × 10^−3^ (±3 10^−4^)	- - - -	- - - -	- - - -	- - - -	- - - -	- - - -	- - - -
10%	0:100 50:50 70:30 90:10	32.01 (±2.6 10^−1^) 98.27 (±6.5 10^−1^) 110.17 (±1.2 10^−1^) 127.27 (±4.1 10^−1^)	32.51 (±1.1) 28.86 (±4.0 10^−1^) 29.67 (±1.3 10^−1^) 28.74 (±2.5 10^−1^)	- - 1.4 × 10^−1^ (±9 10^−2^) 1.2 × 10^−2^ (±5 10^−3^)	5.9 1.4 - -	36.2 1.95 - -	0.31 0.32 - -	14.9 3.14 0.49 0.14	2.75 1.87 1.68 0.14	143 57 32 6	0.6 0.7 0.9 124.7
15%	0:100 50:50 70:30 90:10	30.81 (±3.0 10^−1^) 90.97 (±4.6 10^−1^) 148.21 (±3.0 10^−1^) 292.67 (±4.2 10^−1^)	31.71 (±9.0 10^−1^) 27.46 (±8.0 10^−1^) 25.73 (±7.0 10^−1^) 20.35 (±1.3)	- - - 2.3 × 10^−2^ (±6 10^−3^)	419.1 24.7 0.9 -	0.81 30.43 54.54 -	0.79 0.41 0.22 -	223.4 53.99 2.72 0.25	4.97 3.27 1.33 1.51	- 96 32 7	- 0.3 0.4 2.1

**Table 2:** Average fiber diameter values in the LSL/EC-electrospun nanostructures obtained.

Systems		Fiber Diameter (μm)
8%	50:50 70:30 90:10	0.17 ± 5.7 10^−2^ Beads Beads
10%	50:50 70:30 90:10	0.19 ± 3.4 10^−2^ 0.12 ± 4.1 10^−2^ Beads
15%	50:50 70:30 90:10	0.23 ± 5.2 10^−2^ 0.22 ± 4.8 10^−2^ 0.15 ± 1.1 10^−1^

**Table 3:** Average values of the friction coefficient and wear scar diameters obtained with the dispersions of LSL:EC nanostructures in castor oil acting as lubricants.

Concentration (wt%)	Ratio LSL:EC	Nanostructure Concentration (wt%)	Friction Coefficient (-)	Wear Scar Diameter (μm)
15%	50:50	1020	0.088 ± 2.2 10^−3^ 0.077 ± 3.7 10^−3^	225 ± 12.1 228 ± 14.4
	70:30	10 20 30	0.094 ± 5.1 10^−3^ 0.088 ± 1.5 10^−3^ 0.082 ± 1.9 10^−3^	259 ± 42.3 252 ± 8.3 208 ± 39.8
	90:10	20 30	0.089 ± 4.6 10^−3^ 0.081 ± 5.2 10^−3^	250 ± 24.9 201 ± 6.1
Castor oil			0.071 ± 2.9 10^−3^	523 ± 13.6

## Data Availability

Not applicable.
